# Preclinical Dosimetry for Small Animal Radiation Research in Proton Therapy: A Feasibility Study

**DOI:** 10.14338/IJPT-22-00035.1

**Published:** 2023-04-03

**Authors:** Fatih Biltekin, Christian Bäumer, Johannes Esser, Osamah Ghanem, Gokhan Ozyigit, Beate Timmermann

**Affiliations:** 1 Department of Radiation Oncology, Faculty of Medicine, Hacettepe University, Ankara, Turkey; 2 West German Proton Therapy Centre Essen (WPE), Essen, Germany; 3 West German Cancer Centre (WTZ), Essen, Germany; 4 German Cancer Consortium (DKTK), Heidelberg, Germany; 5 TU Dortmund University, Department of Physics, Dortmund, Germany; 6 Department of Particle Therapy, University Hospital Essen, Essen, Germany

**Keywords:** preclinical dosimetry, 3D printer, small animal phantom, proton therapy

## Abstract

**Purpose:**

To evaluate the feasibility of the three-dimensional (3D) printed small animal phantoms in dosimetric verification of proton therapy for small animal radiation research.

**Materials and Methods:**

Two different phantoms were modeled using the computed-tomography dataset of real rat and tumor-bearing mouse, retrospectively. Rat phantoms were designed to accommodate both EBT3 film and ionization chamber. A subcutaneous tumor-bearing mouse phantom was only modified to accommodate film dosimetry. All phantoms were printed using polylactic-acid (PLA) filament. Optimal printing parameters were set to create tissue-equivalent material. Then, proton therapy plans for different anatomical targets, including whole brain and total lung irradiation in the rat phantom and the subcutaneous tumor model in the mouse phantom, were created using the pencil-beam scanning technique. Point dose and film dosimetry measurements were performed using 3D-printed phantoms. In addition, all phantoms were analyzed in terms of printing accuracy and uniformity.

**Results:**

Three-dimensionally printed phantoms had excellent uniformity over the external body, and printing accuracy was within 0.5 mm. According to our findings, two-dimensional dosimetry with EBT3 showed acceptable levels of γ passing rate for all measurements except for whole brain irradiation (γ passing rate, 89.8%). In terms of point dose analysis, a good agreement (<0.1%) was found between the measured and calculated point doses for all anatomical targets.

**Conclusion:**

Three-dimensionally printed small animal phantoms show great potential for dosimetric verifications of clinical proton therapy for small animal radiation research.

## Introduction

Small animal models, particularly rat or mouse models, are the most widely used tool to evaluate the radiobiological effects of ionizing radiation on tumor models and normal tissue [1, 2]. Preclinical studies also provide a practical avenue to validate the safety of new drugs, combination therapies, or novel treatment modalities before introducing them into clinical practice. However, the translation of the preclinical data into clinical practice is limited because of both the lack of standardization and uncertainties [3–6]. The main causes of the uncertainties can be classified as follows: (1) the lack of awareness of the preclinical scientist about the importance of quality assurance (QA) of the radiotherapy machine and dedicated micro-irradiator, (2) insufficient support from medical physicists and/or dosimetrists, and (3) inadequate equipment or dosimetric system to perform QA tests [2]. In addition to physical parameters, radiobiological experiments are burdened with large systematic uncertainties owing to the variations associated with the biologic response of in vivo models [7]. In the literature, Hackam et al [8] reported that only 28 of 76 highly cited animal studies were continued subsequently in the frame of clinical studies, and 14 of these had contradictory results with respect to the findings in preclinical studies. The absence of standardization (ie, irradiation techniques, reproducibility of the setup, dose calculation, delivery accuracy) is recognized as the main concern for the discrepancy between radiobiological experiments and clinical studies [2–8]. Recently, a variety of dedicated equipment (eg, micro–computed tomography [CT] for both simulation and image guidance, sophisticated treatment planning system (TPS) for dose calculation, micro–animal irradiator) became available to reduce the total uncertainties owing to the irradiation. Nevertheless, either a standard linear accelerator or a particle therapy system is routinely used in many clinics to avoid large capital investments for preclinical research. However, radiotherapy platforms intended for human use may present some challenges (eg, use of a very small field size, effects of the buildup region and a wider penumbra owing to the higher beam energy, lower resolution in dose calculation) compared with dedicated systems. In addition, dose delivery on a small animal scale is not generally verified as part of a QA program in clinical external radiotherapy with photons or protons. In order to verify the dosimetric accuracy of the treatment platform, an independent evaluation of the delivered dose needs to be conducted with homogeneous or zoomorphic small animal phantoms like mouse and rat phantoms. Reports in the literature indicate that 3D-printing technology can be a very effective tool for producing anatomically corrected small animal phantoms [7, 9–13], immobilization devices [14], and QA phantom for preclinical irradiation [15]. However, to the best of our knowledge, no studies evaluate the feasibility of 3D-printed rat and mouse phantoms for the verification of a standard proton therapy system in small animal radiation research. The present study aims to evaluate the feasibility of 3D-printed rat and tumor-bearing mouse phantoms in dosimetric verification of preclinical irradiation with proton therapy.

## Materials and Methods

### Modeling and 3D Printing

**Figure 1.**
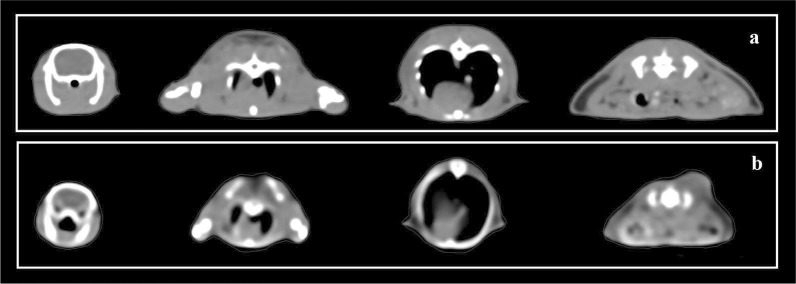
Axial CT images of real (a) rat and (b) subcutaneous tumor-bearing mouse. Abbreviation: CT, computed tomography.

**Figure 2.**
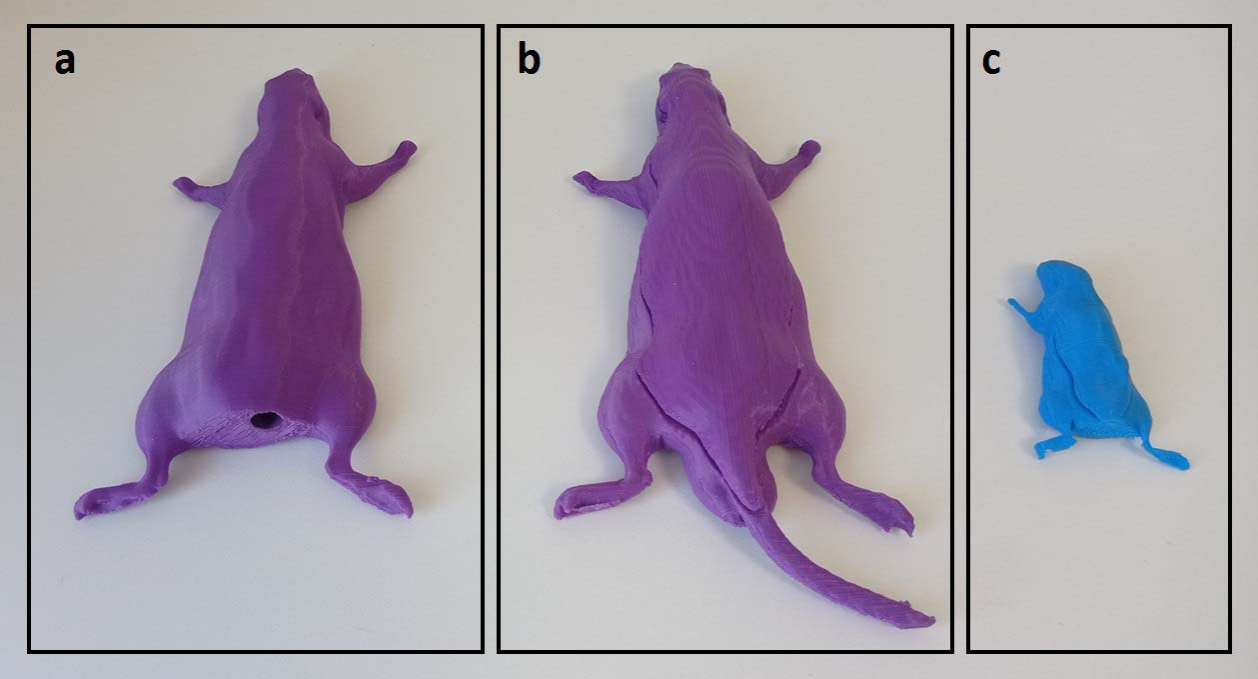
A representative view of 3D printed rat phantoms for (a) point dose measurement, (b) film dosimetry, and (c) subcutaneous tumor-bearing mouse phantom for film dosimetry. Abbreviation: 3D, three-dimensional.

Two different small animal phantoms comprising rat and subcutaneous tumor-bearing mouse phantoms were modeled based on the segmented CT data of a real rat and mouse previously scanned for another radiobiological experiment (**Figure 1**). For a homogeneous phantom design, the external body was segmented in the RayStation TPS (version 10.2, RaySearch Laboratories, Stockholm, Sweden), and the structure set was exported to the 3D Slicer software (version 4.3, open-source software, the Slicer Community, Harvard, Massachusetts) to convert DICOM files to the stereolithographic file format (.stl), which is an openly documented file format to describe the surface of the object for 3D printing and computer-aided manufacturing. The rat and mouse phantoms were then printed in the MakerBot Replicator Z18 3D printer (MakerBot Industries, Brooklyn, New York) using PLA filament, a thermoplastic polyester with a density of 1.25 g/cm^3^ (**Figure 2**). Printing parameters were set as diamond infill pattern, 95% infill percentage, and vertical printing directions to create tissue-equivalent phantoms. Tissue-equivalent rat phantoms were designed to accommodate both a Gafchromic EBT3 film (Ashland Advanced Materials, Bridgewater, New Jersey) and an ionization chamber system. The phantom of the subcutaneous tumor-bearing mouse deviated from the zoomorphic approach regarding a slit to accommodate film dosimetry. In addition to a homogeneous rat phantom, a heterogeneous rat phantom (including lungs modeled as air cavity) was printed to create a simple zoomorphic phantom for film dosimetry. Since the phantoms were printed with non–tissue-equivalent plastic material, the water equivalent ratio (WER) of the material was verified with a multilayer ionization chamber (MLIC) system (Zebra, IBA Dosimetry, Schwarzenbruck, Germany) using a simplistic phantom (dimension: 50 mm × 50 mm × 10 mm) created with the same printing parameters.

### Physical Measurements and Printing Accuracy

The printing accuracy of the phantoms was evaluated through physical measurements using a vernier caliper with a resolution of 0.1 mm. Physical measurements were performed at multiple positions along the prints, and all measurements were compared with the modeled dimensions of the phantoms in TPS. In addition, all phantoms were scanned with the Philips Brilliance Big Bore 16-slice CT simulator (Philips Medical Systems, Cleveland, Ohio) using standard protocols with 100 kVp, 200 mAs, and iterative reconstruction. The reconstructed slice thickness was set as 1 mm for all image sets. After that, the mean Hounsfield unit (HU) values and line profiles for HU in both superior-inferior and left-right direction were analyzed in the TPS to evaluate the uniformity of the phantoms.

### Contouring and Treatment Planning

**Figure 3.**
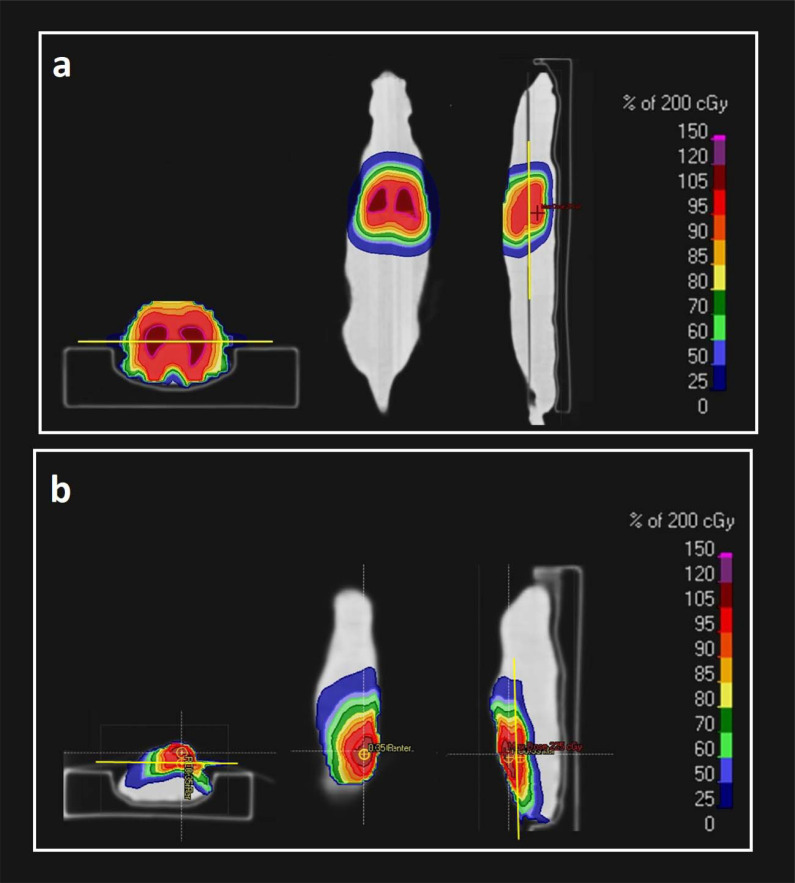
PBS proton therapy plans for (a) total lung irradiation in heterogeneous rat phantom and (b) subcutaneous tumor in mouse phantom. Yellow lines in axial and sagittal images depict the film dosimetry location, and the dose distributions are overlaid as a color wash on CT slices. Abbreviations: CT, computed tomography; PBS, pencil-beam scanning.

In the first part, target volumes for various anatomical sites of therapeutic interest, including whole brain and total lung, were defined on a CT dataset of the rat phantoms (including both tissue-equivalent and zoomorphic phantoms) using the fused real CT dataset of the rat. In the second part, an orthotopic subcutaneous tumor was contoured in the tumor-bearing mouse phantom. In the frame of treatment planning, 5 different treatment plans including 2 plans for point dose and 2 plans for film measurements (whole brain and total lung irradiation)

in the homogeneous rat phantom and one plan for film measurement in the heterogeneous rat phantom (total lung irradiation) were created in the RayStation TPS (**Figure 3a**). In addition, one treatment plan was created for a tumor-bearing mouse phantom as illustrated in **Figure 3b**. All phantoms were immobilized with 3D-printed immobilization devices to easily set the phantom position during both simulation and irradiation. In all treatment plans, a single anterior-posterior treatment field and the pencil-beam scanning (PBS) technique with range shifter were used to create optimal treatment plans. During optimization, range robustness was also applied as ±3.5% to account for the range of uncertainty during conversion from HU to proton stopping power. In the frame of dose prescription, a constant relative biological effectiveness (RBE) of 1.1 was already applied in TPS to correct physical dose, and a 2-Gy(RBE) dose was prescribed for all defined target volumes. The same correction factor was also applied for the physically measured dose to make it comparable with the calculated dose in TPS.

### Dosimetric Measurements

**Figure 4.**
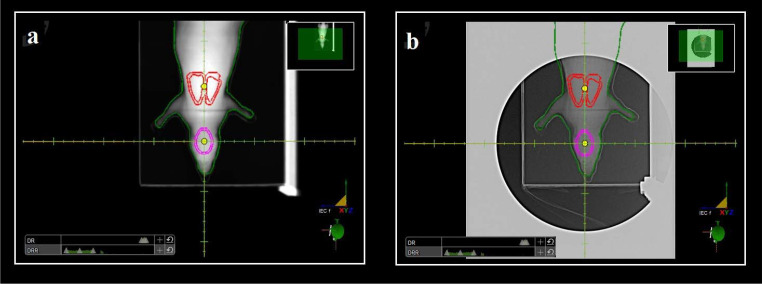
Screenshots of the anterior setup field with segmented external body (green), brain (purple), and lungs (red); (a) transferred from RayStation TPS to imaging software and (b) taken with on-board x-ray imaging before measurement for whole brain irradiation. Abbreviation: TPS, treatment planning system.

All measurements were performed on the IBA ProteusPlus proton therapy system equipped with the IBA universal nozzle (IBA PT, Louvain-La-Neuve, Belgium) installed in the West German Proton Therapy Centre Essen (WPE). For point dose measurements, the CC01 ionization chamber (IBA Dosimetry) with 0.01-cm^3^ sensitive volume was used, and the detector was positioned to the predefined treatment planning position for both whole brain and total lung irradiation. Measurements were repeated 2 times to evaluate the reproducibility of the measured dose values. For film dosimetry, radiochromic EBT3 films (Ashland Gafchromic, Bridgewater, New Jersey) were rigidly fixed within the defined regions of the rat and mouse phantoms in a coronal plane, thereby enabling the acquisition of 2-dimensional (2D) spatial- and depth-dose information, simultaneously. For all cases, on-board portal x-ray imaging was used to localize the isocenter of the treatment plans (with ionization chamber and without film dosimetry) prior to each irradiation (**Figure 4**).

During the evaluation, point dose values calculated in the TPS were compared with measured point dose values obtained with the ionization chamber. For 2D dosimetry, Gafchromic films were scanned with an EPSON 10000XL flatbed scanner (Epson America, Long Beach, California) after 24 hours. The 2D γ-analysis methods available in the PTW VeriSoft software (version 4.1, PTW, Freiburg, Germany) were used to evaluate the agreement between measured and calculated dose maps. As evaluation criteria, 2-mm distance to agreement (DTA)/2% dose differences (DD) and 3-mm DTA/3% DD were used. Doses below 10% of the prescription dose were disregarded, and the threshold for γ–passing rate was set at 95% as a protocol value (minor variation ≥90%).

## Results

### Physical Measurements and Printing Accuracy

**Figure 5.**
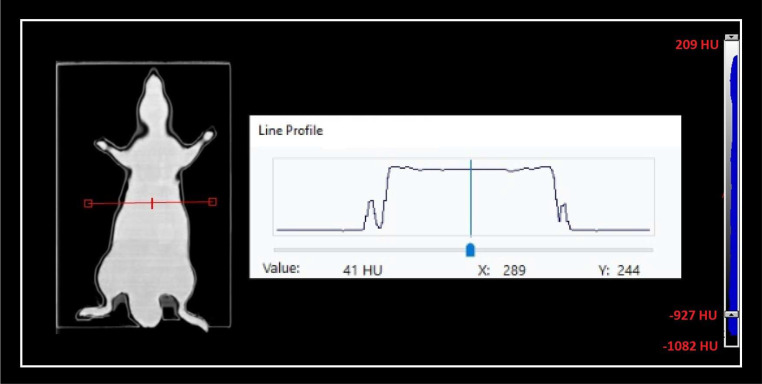
A representative horizontal line profile of (HU) value for homogeneous rat phantom. Abbreviation: HU, Hounsfield units.

The maximum measured differences between the modeled and the printed dimensions of the phantoms were within 0.5 mm (±0.1-mm resolution of the vernier caliper). Printed dimensions of the external body of the phantoms were on average 0.4 mm (range, 0.3-0.5 mm) greater than the modeled phantom. In contrast to the external body, dimensions of the created cavities for both ionization-chamber and film-dosimeter channels were on average 0.2 mm (range, 0.0-0.3 mm) smaller than the modeled ones. In addition, CT scans of the 3D-printed homogeneous mouse phantoms had excellent uniformity over the external body. There was not any region containing unwanted air cavities or high-density areas as illustrated in **Figure 5**. The mean HU values averaged over all the voxels for the homogeneous rat phantoms, excluding detector cavities, were between −0.49 and −25.35 HU. Similarly, the mean HU value for the tumor-bearing mouse phantom was −22.42 HU.

### Dosimetric Measurements

**Figure 6.**
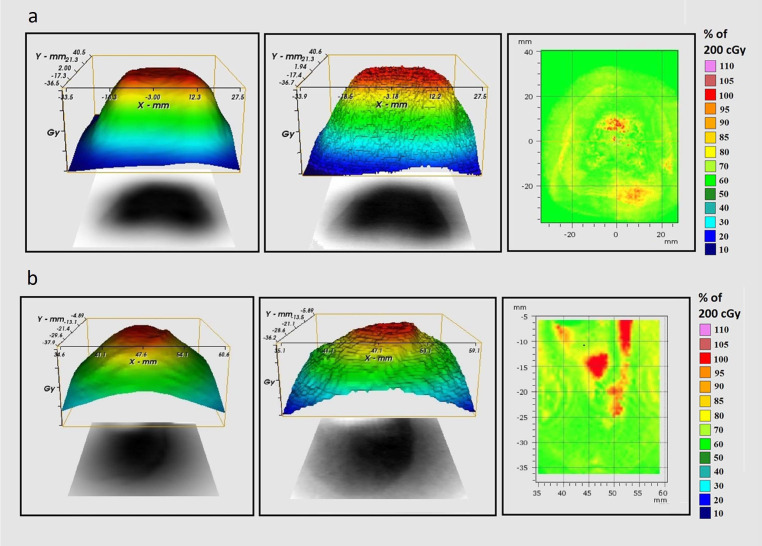
Gamma analysis for (a) total lung irradiation in heterogeneous rat phantom and (b) subcutaneous tumor plan in mouse phantom. Dose maps calculated in TPS (left), dose maps measured with EBT3 Gafchromic film (middle), and γ distribution for 3-mm DTA/3% DD (right). Abbreviations: DD, dose differences; DTA, distance to agreement; TPS, treatment planning system.

According to the MLIC measurements, the 3D-printed material with defined printing parameters (diamond infill pattern, 95% infill percentage, and vertical direction) was found to have WER = 1.0. Therefore, a density correction was not applied during the dose calculation. In terms of point dose measurements, dose values measured with the ionization chamber were found as 2.039 Gy(RBE) (calculated in TPS, 2.040 Gy[RBE]) and 1.989 Gy(RBE) (calculated in TPS, 1.988 Gy[RBE]) for whole brain and total lung irradiation, respectively. In terms of film dosimetry, as also illustrated in **Table 1**, γ–passing rates for all measurements were found higher than 90% except for the whole brain irradiation (γ–passing rate, 89.8%). Gamma analyses for total lung irradiation in rat phantom and subcutaneous tumor irradiation in mouse phantoms are illustrated in **Figure 6a** and **6b**, respectively.

**Table 1.** Gamma analysis of EBT3 Gafchromic film measurement.

	**Gamma passing rate (%)**
**Treatment plans (target)**	**Film dosimetry**
Whole brain	98.2^a^/89.8^b^
Total lung (hom)^†^	98.8^a^/90.3^b^
Total lung (het)^†^	99.2^a^/91.0^b^
Subcutanous tumor	93.8^a^/90.9^b^

**Abbreviations:** hom, treatment plan created in homogeneous phantom; het, treatment plan created in zoomorphic phantom.

a Evaluation criteria: 3-mm DTA/3% DD, 10% dose threshold.

b Evaluation criteria: 2-mm DTA/2% DD, 10% dose threshold.

## Discussion

We have presented the feasibility of 3D-printed small animal phantoms for the dosimetric verification of small animal radiation research in clinically available proton therapy. According to our findings, calculated and measured point doses were always within 0.1%, and the minimum γ–passing rate for film dosimetry was 89.8% for all defined scenarios in rat phantoms and 90.8% for the tumor-bearing mouse phantom using 2-mm DTA/2% DD. Although the higher resolution of the Gafchromic films makes them attractive for 2D dose measurement, the calibration of film dosimetry is generally a tedious and time-consuming procedure limiting the widespread use of it in clinical practice [16]. In addition, the film response can be significantly affected by several factors such as the lateral response artifact (or parabola effect) caused by the nonuniform scanner response along the scanned axis [17], linear energy transfer (LET)-dependent film response at low-energy proton and carbon beams [18, 19], and the film orientation relative to the proton beam direction. Regarding the latter effect, using films parallel to the beam central axis may cause an artifact in the dose distribution owing to the air gap between film and phantom [16]. Therefore, all these limitations need to be addressed before considering film dosimetry as a verification tool for accurate measurement of absolute doses, and even relative metrics. Moreover, the use of small fields in proton therapy is another concern that needs to be considered. Detector size, aperture scattering, and disequilibrium in small fields have significant impact on QA results [20, 21]. In addition, the sharp lateral dose falloff is one of the most demanding issues in proton therapy, especially for small or stereotactical fields to spare normal tissue as much as possible. To overcome this problem, Bäumer et al [22] showed that the use of a collimating aperture mounted between the range shifters and the patient in PBS technique could improve the lateral dose gradient. However, the present study did not focus on the sparing of normal tissue and could be extended in this regard.

Another noteworthy point is that despite the importance of accurate dose delivery in preclinical radiation research, verification of the delivered dose still has not been the subject of many preclinical studies. Therefore, the translation of the preclinical data into clinical practice is generally limited because of both the lack of standardization in methodology and uncertainties, especially in dosimetry [3–6]. Inadequate equipment or dosimetric systems are defined as one of the most challenging issues in preclinical dosimetry. To overcome this problem, 3D-printing technology has been proposed as a promising solution to create a cost-effective and reliable equipment or tool in radiotherapy facilities including compensator materials for proton therapy [23, 24], a bolus [25–29], QA phantoms for external-beam radiotherapy [30–34], and applicators and auxiliary equipment for brachytherapy facilities [35–39]. Similarly, small animal phantoms have also been created to assess the accuracy of several steps in photon-based preclinical radiation research: CT scanning, treatment planning, image guidance, and treatment delivery [9–13]. Biglin et al [40] undertook a dosimetry audit of Xstrahl small animal radiation research platforms (SARRPs), installed at 7 centres in the United Kingdom, using 3D-printed murine phantoms. The audit demonstrated that further work on preclinical radiotherapy QA processes with 3D-printed phantoms is merited. In addition, in the literature, the printing accuracy of the 3D-printed phantom was evaluated in detail by different researchers [10, 13]. Esplen et al [10] reported that the micro–CT imaging of the 3D-printed mouse phantom revealed an excellent uniformity over the printed product and agreed with the designed model. In another study, Price et al [13] also evaluated the quality of the geometric agreement as the DTA of the registered body surface of the reference-CT and phantom-CT images. They reported that the body surface of the printed phantom agreed within 39%, 74%, and 90% for 0.5-, 1.0-, and 1.5- mm DTA values, respectively. In addition, they provided their design and full methodology as an open source to encourage the preclinical radiobiology community and to adopt a common QA standard. In the present study, the printed dimensions of the external body of the phantoms were on average 0.4 mm (range, 0.3-0.5 mm) greater than the 3D models, and the dimensions of the created cavities for both ionization chamber and film dosimeter were on average 0.2 mm (range, 0.0-0.3 mm) smaller than the modeled ones. The reason could be associated with the comparison of the physical measurements with the values directly taken from TPS. However, the volume reconstruction algorithm or automatic smoothing tool used in the 3D Slicer program might also cause a slight difference in phantom dimensions during the modeling of the external body surface [41]. In addition, printing parameters (eg, printing resolution, infill pattern, printing direction) might also affect the dimensions of the printed product. The effect of the printing parameters could be a subject of further investigation to improve the quality of 3D-printed small animal phantoms.

This study only focused on the feasibility of simplistic 3D-printed small animal phantoms for the dosimetric verification of the clinically available proton therapy system in radiobiological experiments. However, in the future, anatomically corrected small animal phantom printed with a mimetic material or using printable/chemically engineered resin mimicking full anatomy, including muscle and fat, may be a promising solution for advanced verification of the delivered dose in realistic anatomy [10]. Further, as performed by Biglin et al. [40], a multi-centre dosimetry audit can be performed to verify the plan delivery in clinical particle therapy beam for small animal radiation research. In addition, a dedicated version of the TPS system compatible to clinically available treatment platforms for preclinical research is currently available to meet the needs (eg, smaller voxels for specimen modeling and dose computations down to 0.1-mm resolution) in the field of small animal irradiation research. However, the main idea behind the proposed method of the present study is the simplification and cost-effectiveness of the current approach for easily adopting it into all radiobiology laboratories and clinics using current technology and available equipment.

## Conclusion

Three-dimensionally printed small animal phantoms enable the dosimetric verifications of a clinical proton therapy system for radiobiological experiments. Therefore, 3D-printed rat and mouse models show great potential in the adoption of a common QA standard for preclinical studies as they can be easily and reproducibly manufactured using current technology. In addition, as also pointed out by Biglin et al [2], before conducting animal research, as researchers, we are ethically obliged to ensure that the data from each animal used in a radiobiological experiment support the purpose of the study and contribute to the progress of novel therapeutic strategies. Hence, the phantoms also allow training for radiotherapy planning and independent end-to-end verification of the dose-delivery protocol to evaluate the feasibility of irradiation without sacrificing animals.
